# The uniqueness of a nonlinear diffusion equation related to the *p*-Laplacian

**DOI:** 10.1186/s13660-017-1596-4

**Published:** 2018-01-05

**Authors:** Huashui Zhan

**Affiliations:** School of Applied Mathematics, Xiamen University of Technology, Xiamen, 361024 P.R. China

**Keywords:** 35L65, 35L85, 35R35, *p*-Laplacian, diffusion coefficient, boundary value condition, uniqueness

## Abstract

Consider a nonlinear diffusion equation related to the *p*-Laplacian. Different from the usual evolutionary *p*-Laplacian equation, the equation is degenerate on the boundary due to the fact that the diffusion coefficient is dependent on the distance function. Not only the existence of the weak solution is established, but also the uniqueness of the weak solution is proved.

## Introduction and the main results

Recently, we noticed that Benedikt *et al.* [1] had studied the equation

1.1 $$ {u_{t}} = \operatorname{div} \bigl({ \vert {\nabla u} \vert ^{p - 2}}\nabla u\bigr) + q(x)u^{\gamma} ,\quad (x,t) \in{Q_{T}\Omega\times (0,T)} , $$

and shown that the uniqueness of the solutions of equation (1.1) is not true. Here, Ω is an open bounded domain with a smooth boundary, $0<\gamma<1$, $p>1$, $q(x)\in C^{1}(\overline{\Omega})$, $q(x)\geq0$ and there exists at least a point $x_{0}\in\Omega$, $q(x_{0})>0$. This comes more or less as a surprise. In general, we may think that the source time $q(x)u^{\gamma}$ only affects the existence of the weak solutions. At the same time, in [2], we have considered the following equation:

1.2 $$ {u_{t}} = \operatorname{div} \bigl(\rho^{\alpha}{ \vert { \nabla u} \vert ^{p - 2}}\nabla u\bigr) + f(u,x,t), \quad (x,t) \in{Q_{T}}, $$

and we have shown that the uniqueness of the weak solution is true when $f(u,\cdot, \cdot)$ is a Lipschitz function, here $\alpha>0$, $\rho(x) = \operatorname{dist} (x,\partial\Omega)$ is the distance function from the boundary. Certainly, since $0<\gamma<1$ in equation (1.1), $f(u,x,t)=q(x)u^{\gamma}$ is not a Lipschitz function about the variable *u*. Consequently, the results in [1] and [2] are compatible.

If $\alpha=0$, there are a great deal of papers devoted to equations (1.2), many of them are important and interesting. But it is impossible to list all these papers, and we only list a few of them [3–7] here.

In this paper, we assume that $q(x)\in C^{1}(\overline{\Omega})$. We will consider a nonlinear convection-diffusion equation related to the *p*-Laplacian,

1.3 $$ u_{t} = \operatorname{div} \bigl(\rho^{\alpha} \vert \nabla u \vert ^{p - 2}\nabla u\bigr) + \sum_{i = 1}^{N} \frac{\partial b_{i}(u)}{\partial x_{i}} +q(x)|u|^{\gamma-1}u, \quad (x,t) \in Q_{T}, $$

where $0<\gamma<1$. The initial value condition

1.4 $$ u(x,t)=u_{0}(x),\quad x\in\Omega, $$

is always necessary. Different from the usual evolutionary *p*-Laplacian equation or equation (1.1), an obvious feature of equations (1.2), (1.3) lies in that the diffusion coefficient $\rho ^{\alpha}$ depends on the distance to the boundary. By this feature, instead of the usual boundary value condition

1.5 $$ u(x,t)=0,\quad (x,t)\in\partial\Omega\times(0,T), $$

only a partial boundary condition,

1.6 $$ u(x,t)=0, \quad (x,t)\in\Sigma_{p}\times(0,T), $$

should be imposed generally, where $\Sigma_{p}\subseteq\partial\Omega $ is a relatively open subset in *∂*Ω. One can refer to our previous work [2, 8].

Since equation (1.3) is a nonlinear equation, it is difficult to depict $\Sigma_{p}$ as the linear degenerate parabolic equation by the Fichera function. The main aim of this paper is to prove the uniqueness of the solutions without any boundary value condition.

In the first place, since we had known the interesting result of [1] (*i.e.* the nonuniqueness of the weak solution of equation (1.1)), we should clarify why the uniqueness of the weak solutions of equation (1.3) can be obtained. Let us introduce some basic functional spaces. For every fixed $t\in[0, T)$, we define the Banach space

$$\begin{aligned}& V_{t}(\Omega) =\bigl\{ u(x,t) : u(x,t)\in L^{2}(\Omega)\cap W^{1,1}_{0}(\Omega ), \bigl\vert \nabla u(x,t) \bigr\vert ^{p}\in L^{1} (\Omega)\bigr\} , \\& \|u\|_{V_{t}(\Omega)} = \|u\|_{2,\Omega} + \|\nabla u\|_{p,\Omega} , \end{aligned}$$

and we denote by $V'_{t}(\Omega)$ its dual. Also, we denote the Banach space

$$\textstyle\begin{cases} \mathbf{W}(Q_{T}) = \{u : [0,T]\rightarrow V_{t}(\Omega)|u\in L^{2}(Q_{T}),|\nabla u|^{p} \in L^{1}(Q_{T}), u = 0\mbox{ on }\Gamma=\partial \Omega\}, \\ \|u\|_{\mathbf{W}(Q_{T})} = \|\nabla u\|_{p,Q_{T}} + \|u\|_{2,Q_{T}} , \end{cases} $$

and we denote by $\mathbf{W}'(Q_{T})$ its dual. According to Antontsev-Shmarev [9], we know

$$w\in\mathbf{W}'(Q_{T})\quad \Longleftrightarrow\quad \textstyle\begin{cases} w=w_{0}+\sum_{i=1}^{N}D_{i}w_{i},\quad w_{0}\in L^{2}(Q_{T}), w_{i}\in L^{p'}(Q_{T}), \\ \forall\phi\in\mathbf{W}(Q_{T}), \quad \langle\!\langle w,\phi\rangle\!\rangle=\iint_{Q_{T}} (w_{0}\phi+\sum_{i}^{N}w_{i}D_{i}\phi )\,dx\,dt. \end{cases} $$

The norm in $\mathbf{W}'(Q_{T})$ is defined by

$$\|v\|_{\mathbf{W}'(Q_{T})}= \sup\bigl\{ \langle\!\langle v,\phi\rangle\! \rangle|\phi\in\mathbf {W(Q_{T})},\|\phi\|_{\mathbf{W}(Q_{T})}\leq1\bigr\} . $$

Basing on these functional spaces, we can give the definition of the weak solution.

### Definition 1.1

A nonnegative function $u(x,t)$ is said to be a weak solution of equation (1.3) with the initial value (1.4), if

1.7 $$ u \in{L^{\infty}}({Q_{T}}),\qquad {\rho^{\alpha}} { \vert {\nabla u} \vert ^{p}} \in{L^{1}}({Q_{T}}), \qquad u_{t}\in\mathbf{W}'(Q_{T}), $$

and, for any function $\varphi \in L^{\infty}(0,T; W_{0}^{1.p}(\Omega ))\cap\mathbf{W}(Q_{T})$,

1.8 $$\begin{aligned}& \langle\!\langle u_{t},\varphi\rangle\!\rangle+ \iint_{Q_{T}}\Biggl(\rho^{\alpha} \vert \nabla u \vert ^{p - 2}\nabla u \cdot\nabla\varphi + \sum_{i = 1}^{N} b_{i}(u) \cdot\varphi_{x_{i}}\Biggr)\,dx\,dt \\& \quad = \iint _{Q_{T}}q(x)|u|^{\gamma-1}u\varphi \,dx \,dt. \end{aligned}$$

The initial value is satisfied in the sense that

1.9 $$ \lim_{t\rightarrow0} \int_{\Omega} \bigl\vert u(x,t)-u_{0}(x) \bigr\vert \, dx=0. $$

The most important of Definition 1.1 lies in $u_{t}\in\mathbf {W}'(Q_{T})$. Once the weak solution comes with this property, then we have Lemma 3.1 below, and just by this lemma, we can prove the uniqueness. By comparing the analysis in [1], we know the weak solution defined in [1] does not have this property.

Second, we introduce the existence result.

### Theorem 1.2

*If* $p>1$, $0<\gamma<1$, $b_{i}(s)$ *is a* $C^{1}$ *function*, *and*

1.10 $$ {u_{0}} \in{L^{\infty}}(\Omega),\qquad {\rho^{\alpha}} { \vert {\nabla {u_{0}}} \vert ^{p}} \in{L^{1}}( \Omega), $$

*then equation* (1.1) *with initial value* (1.4) *has a weak solution*.

Last but not least we will prove the following local stability.

### Theorem 1.3

*Let* $p>1$, $\gamma>0$, $b_{i}(s)$ *be a Lipschitz function*. *If* *u*, *v* *are two solutions of equation* (1.3) *with the initial values* $u_{0}(x)$, $v_{0}(x)$, *respectively*, *then there exists a positive constant* $\beta\geq\max\{\frac{p-\alpha}{p-1}, 2, \alpha p \}$ *such that*

1.11 $$ \int_{\Omega}\rho^{\beta} \bigl\vert u(x,t)-v(x,t) \bigr\vert ^{2}\, dx\leq c \int_{\Omega }\rho^{\beta} \bigl\vert u_{0}(x)-v_{0}(x) \bigr\vert ^{2}\, dx. $$

*In particular*, *for any small enough constant* $\lambda>0$,

1.12 $$ \int_{\Omega_{\lambda}} \bigl\vert u(x,t)-v(x,t) \bigr\vert ^{2}\, dx\leq c\lambda^{-\beta } \int_{\Omega} \bigl\vert u_{0}(x)-v_{0}(x) \bigr\vert ^{2}\, dx. $$

Here, $\Omega_{\lambda}=\{x\in\Omega: \operatorname{dist}(x,\partial\Omega )>\lambda\}$, by the arbitrariness of *λ*, we have the uniqueness of the solution. This conclusion implies that the degeneracy of the diffusion coefficient can take place of the usual boundary value condition.

We would like to suggest that, if $\rho^{\alpha}$ is substituted by a nonnegative diffusion coefficient $a(x)\in C^{1}(\overline{\Omega})$ with

$$a(x)|_{x\in\Omega}>0,\qquad a(x)|_{x\in\partial\Omega}=0, $$

a similar conclusion to Theorem 1.3 is still true. For some special cases, one can see our recent work [10]. Actually, we had used some ideas of [10] to prove Theorem 1.3.

This paper is arranged as follows. In Section 1, we give the basic definition and introduce the main results. In Section 2, we prove the existence of the solution to equation (1.1) with initial value (1.4). In Section 3, we prove Theorem 1.3 and obtain the uniqueness of the solution.

## The weak solutions dependent on the initial value

We consider the weak solution of the initial value problem for equation (1.3) in this section. It is supposed that $u_{0}$ satisfies

$$u_{0}\in L^{\infty}(\Omega), \qquad { \vert { \nabla{u_{0}}} \vert ^{p}} \in{L^{1}}(\Omega). $$

Let ${u_{\varepsilon,0}} \in{C^{\infty}_{0} }(\Omega)$ and $\rho _{\varepsilon}^{\alpha}{ \vert {\nabla{u_{\varepsilon,0}}} \vert ^{p}}\in {L^{1}}(\Omega)$ be uniformly bounded, and ${u_{\varepsilon,0}}$ converges to $u_{0}$ in $W_{0}^{1,p}(\Omega)$. Here ${\rho_{\varepsilon}} = \rho\ast\delta_{\varepsilon}+ \varepsilon, \varepsilon > 0$, $\delta_{\varepsilon}$ is the mollifier as usual.

By the results of [11, Section 8], we have the following important lemma.

### Lemma 2.1

*If* $u_{\varepsilon}\in L^{\infty}(0,T;L^{2}(\Omega ))\cap\mathbf{W}(Q_{T})$, $\| u_{\varepsilon t}\|_{\mathbf {W}'(Q_{T})}\leq c$, $\|\nabla(|u_{\varepsilon}|^{q-1}u_{\varepsilon })\|_{p,Q_{T}}\leq c$, *then there is a subsequence of* $\{u_{\varepsilon }\}$ *which is relatively compact in* $L^{s}(Q_{T})$ *with* $s\in(1,\infty )$. *Here* $q\geq1$.

We now consider the following regularized problem:

2.1 $$\begin{aligned}& u_{\varepsilon t} - \operatorname{div} \bigl(\rho^{\alpha}_{\varepsilon}\bigl( \vert \nabla{u_{\varepsilon}} \vert ^{2}+\varepsilon \bigr)^{\frac{p - 2}{2}}\nabla{u_{\varepsilon}}\bigr) - \sum _{i = 1}^{N} \frac{\partial b_{i}(u_{\varepsilon})}{\partial x_{i}} \\& \quad = q(x)|u_{\varepsilon}|^{\gamma-1}u_{\varepsilon}, \quad (x,t)\in{Q_{T}}, \end{aligned}$$

2.2 $$\begin{aligned}& {u_{\varepsilon}}(x,t) = 0, \quad (x,t) \in\partial\Omega \times (0,T), \end{aligned}$$

2.3 $$\begin{aligned}& {u_{\varepsilon}}(x,0) = {u_{\varepsilon,0}}(x), \quad x\in\Omega , \end{aligned}$$

since $0<\gamma<1$, it is well known that the above problem has an unique classical solution [12, 13].

By the maximum principle, there is a constant *c* only dependent on ${ \Vert {{u_{0}}} \Vert _{{L^{\infty}}(\Omega)}}$ but independent on *ε*, such that

2.4 $$ { \Vert {{u_{\varepsilon}}} \Vert _{{L^{\infty}}({Q_{T}})}} \leqslant c. $$

Multiplying (2.1) by $u_{\varepsilon}$ and integrating it over $Q_{T}$, we get

$$\begin{aligned}& \frac{1}{2} \int_{\Omega}u_{\varepsilon}^{2}\, dx + \iint_{Q_{T}} \rho_{\varepsilon}^{\alpha}\bigl(|\nabla u_{\varepsilon}|^{2}+\varepsilon\bigr)^{\frac{p-2}{2}}|\nabla u_{\varepsilon}|^{2}\,dx\,dt \\& \qquad {}+ \sum_{i = 1}^{N} \iint_{Q_{T}}u_{\varepsilon}\frac{\partial {b_{i}}(u_{\varepsilon})}{\partial x_{i}} \,dx\,dt \\& \quad = \frac{1}{2} \int_{\Omega}u_{0}^{2}\, dx+ \iint_{Q_{T}}q(x)|u_{\varepsilon}|^{\gamma-1}u_{\varepsilon}\,dx\,dt. \end{aligned}$$

By the fact

$$\begin{aligned} \begin{aligned} \sum_{i = 1}^{N} \iint_{{Q_{T}}} {{u_{\varepsilon}} {\frac {{\partial{b_{i}}({u_{\varepsilon}})}}{{\partial{x_{i}}}}} \, {{d}}x\, {d}t} &= - \sum_{i = 1}^{N} { \iint_{{Q_{T}}} {\frac{{\partial {u_{\varepsilon}}}}{{\partial{x_{i}}}}{b_{i}}({u_{\varepsilon}}) \, {{d}}x\, {d}t}} \\ &= - \sum_{i = 1}^{N} { \int_{\Omega}{\frac{\partial}{{\partial{x_{i}}}} \int_{0}^{{u_{\varepsilon}}} {{b_{i}}(s)\, {d}s\, {d}x} } } = 0, \end{aligned} \end{aligned}$$

then

2.5 $$ \frac{1}{2} \int_{\Omega}{u_{\varepsilon}^{2}\,{d}x} + \iint _{{Q_{T}}} {\rho_{\varepsilon}^{\alpha}\bigl(|\nabla u_{\varepsilon}|^{2}+\varepsilon\bigr)^{\frac{p-2}{2}}|\nabla u_{\varepsilon}|^{2}{\, {d}}x\, {d}t} \leqslant c. $$

It is also easy to show that

2.6 $$ \iint_{{Q_{T}}} \rho^{\alpha}|\nabla u_{\varepsilon}|^{p} \,dx\,dt\leqslant c \iint_{{Q_{T}}} \rho_{\varepsilon}^{\alpha}|\nabla u_{\varepsilon}|^{p}\,dx\,dt\leqslant c. $$

Now, for any $v\in\mathbf{W}(Q_{T})$, $\|v\|_{W(Q_{T})}=1$,

2.7 $$\begin{aligned} \langle u_{\varepsilon t}, v\rangle =&- \iint_{Q_{T}}\rho_{\varepsilon}^{\alpha}\bigl(|\nabla u_{\varepsilon}|^{2} +\varepsilon\bigr)^{\frac{p-2}{2}}\nabla u_{\varepsilon}\cdot\nabla v \,dx\,dt \\ &{}- \iint_{Q_{T}}\frac{\partial v}{\partial x_{i}}{b_{i}}({u_{\varepsilon}})\,dx\,dt+ \iint_{Q_{T}}q(x)|u_{\varepsilon}|^{\gamma-1}u_{\varepsilon}v\, dx\, dt, \end{aligned}$$

by Young inequality, we can show that

$$\bigl\vert \langle u_{\varepsilon t}, v\rangle \bigr\vert \leq c\biggl[ \iint_{{Q_{T}}} \rho_{\varepsilon }^{\alpha} \vert \nabla u_{\varepsilon} \vert ^{p}\,dx\,dt+ \iint_{{Q_{T}}} \bigl( \vert v \vert ^{p}+ \vert \nabla v \vert ^{p}\bigr)\,dx\,dt+1\biggr]\leq c, $$

then

2.8 $$ \Vert u_{\varepsilon t} \Vert _{\mathbf{W}'(Q_{T})}\leq c. $$

Now, let $\varphi\in C_{0}^{1}(\Omega)$, $0\leq\varphi\leq1$ such that

$$\varphi|_{\Omega_{2\lambda}}=1,\qquad \varphi|_{\Omega\setminus \Omega_{\lambda}}=0. $$

Then

$$\bigl\vert \bigl\langle (\varphi u_{\varepsilon})_{t}, v\bigr\rangle \bigr\vert = \bigl\vert \langle \varphi u_{\varepsilon t}, v\rangle \bigr\vert \leq \bigl\vert \langle u_{\varepsilon t}, v\rangle \bigr\vert , $$

we have

2.9 $$\begin{aligned}& \bigl\Vert \bigl(\varphi(x) u\bigr)_{\varepsilon t} \bigr\Vert _{\mathbf{W}'(Q_{T})}\leq \Vert u_{\varepsilon t} \Vert _{\mathbf{W}'(Q_{T})}\leq c, \end{aligned}$$

2.10 $$\begin{aligned}& \iint_{Q_{T}} \bigl\vert \nabla(\varphi u_{\varepsilon}) \bigr\vert ^{p}\,dx\,dt\leq c(\lambda ) \biggl(1+ \int_{0}^{T} \int_{\Omega_{\lambda}}|\nabla u_{\varepsilon}|^{p} \,dx\,dt \biggr)\leq c(\lambda), \end{aligned}$$

and so

2.11 $$ \bigl\Vert \nabla(\varphi u_{\varepsilon}) \bigr\Vert _{p,Q_{T}}\leq c. $$

By Lemma 2.1, $\varphi u_{\varepsilon}$ is relatively compact in $L^{s}(Q_{T})$ with $s\in(1,\infty)$. Then $\varphi u_{\varepsilon}\rightarrow\varphi u$ a.e. in $Q_{T}$. In particular, due to the arbitrariness of *λ*, $u_{\varepsilon}\rightarrow u$ a.e. in $Q_{T}$.

Hence, by (2.4), (2.7), there exists a function *u* and an *n*-dimensional vector function $\vec{\zeta}= ({\zeta_{1}}, \ldots,{\zeta_{n}})$ satisfying

$$u \in L^{\infty}(Q_{T}),\qquad \vert {\vec{\zeta}} \vert \in{L^{\frac{p}{{p - 1}}}}({Q_{T}}), $$

and

$$\begin{aligned}& {u_{\varepsilon}} \rightharpoonup^{*} u,\quad \text{in } {L^{\infty}(Q_{T})}, \qquad u_{\varepsilon}\rightarrow u,\quad \text{a.e. in } Q_{T}. \\& \rho_{\varepsilon}^{\alpha}{ \vert {\nabla{u_{\varepsilon}}} \vert ^{p - 2}} {u_{\varepsilon x_{i}} } \rightharpoonup\zeta_{i}\quad \text{in } {L^{\frac{p}{p-1}}}({Q_{T}}). \end{aligned}$$

In order to prove that *u* satisfies equation (1.3), we notice that, for any function $\varphi \in C_{0}^{\infty}({Q_{T}})$,

2.12 $$\begin{aligned}& \iint_{Q_{T}} \Biggl(-u_{\varepsilon}\varphi_{t} + \rho_{\varepsilon}^{\alpha}\bigl(|\nabla u_{\varepsilon}|^{2}+ \varepsilon \bigr)^{\frac{p-2}{2}}\nabla u_{\varepsilon}\cdot\nabla\varphi + \sum_{i = 1}^{N} b_{i}(u_{\varepsilon}) \cdot\varphi_{x_{i}}\Biggr)\,dx\,dt \\& \quad = \iint_{Q_{T}}q(x)|u_{\varepsilon}|^{\gamma-1}u_{\varepsilon}\varphi \,dx\,dt, \end{aligned}$$

and $u_{\varepsilon} \rightarrow u$ is almost everywhere convergent, so $b_{i}(u_{\varepsilon})\rightarrow b_{i}(u)$, $|u_{\varepsilon}|^{\gamma -1}u_{\varepsilon}\rightarrow|u|^{\gamma-1}u$. Then

2.13 $$\begin{aligned}& \iint_{{Q_{T}}} \Biggl(\frac{\partial u}{\partial t}\varphi + \vec{\varsigma}\cdot\nabla\varphi + \sum_{i = 1}^{N} b_{i}(u) \cdot\varphi_{x_{i}}\Biggr)\,dx\,dt \\& \quad = \iint _{Q_{T}}q(x)|u|^{\gamma-1}u\varphi \,dx \,dt. \end{aligned}$$

Now, if we can prove that

2.14 $$ \iint_{Q_{T}} \rho^{\alpha} \vert \nabla u \vert ^{p - 2}\nabla u \cdot\nabla\varphi_{1} \, dx\, dt = \iint_{Q_{T}} \vec{\zeta}\cdot\nabla \varphi_{1} \,dx\,dt $$

for any function $\varphi_{1} \in C_{0}^{\infty}({Q_{T}})$, then *u* satisfies equation (1.3). In what follows, we will use a similar method to that in [14] to prove (2.14).

Let $0 \leqslant\psi \in C_{0}^{\infty}({Q_{T}})$ and $\psi=1$ in $\operatorname{supp}\varphi_{1}$. Let $v \in {L^{\infty}}({Q_{T}})$, ${\rho^{\alpha}}{ \vert {\nabla v} \vert ^{p}} \in {L^{1}}({Q_{T}})$. It is well known that

2.15 $$ \iint_{{Q_{T}}} \psi\rho_{\varepsilon}^{\alpha}\bigl({ \vert {\nabla {u_{\varepsilon}}} \vert ^{p - 2}}\nabla{u_{\varepsilon}} - { \vert {\nabla v} \vert ^{p - 2}}\nabla v\bigr) \cdot( \nabla{u_{\varepsilon}} - \nabla v)\,dx\,dt \geqslant0. $$

By choosing $\varphi = \psi{u_{\varepsilon}}$ in (2.12), we can obtain

2.16 $$ \begin{aligned}[b] & \iint_{{Q_{T}}} {\psi\rho_{\varepsilon}^{\alpha}\bigl(| \nabla u_{\varepsilon}|^{2}+\varepsilon\bigr)^{\frac{p-2}{2}}}| \nabla{u_{\varepsilon}}|^{2}\,dx\,dt \\ &\quad = \frac{1}{2} \iint_{{Q_{T}}} {\psi_{t}}u_{\varepsilon}^{2} \,dx\,dt - \iint _{{Q_{T}}} \rho_{\varepsilon}^{\alpha}{u_{\varepsilon}} \bigl(|\nabla u_{\varepsilon}|^{2}+\varepsilon\bigr)^{\frac{p-2}{2}} \nabla{u_{\varepsilon}} \cdot\nabla\psi \,dx\,dt \\ &\qquad {}- \sum_{i = 1}^{N} \iint_{Q_{T}} b_{i}(u_{\varepsilon}) (u_{\varepsilon{x_{i}}}\psi + u_{\varepsilon}\psi_{x_{i}})\,dx\,dt + \iint_{Q_{T}}q(x)|u_{\varepsilon}|^{\gamma-1}u_{\varepsilon}\varphi \,dx\,dt. \end{aligned} $$

Noticing that, when $p\geq2$,

$$\begin{aligned}& \bigl( \vert \nabla u_{\varepsilon} \vert ^{2}+\varepsilon \bigr)^{\frac{p - 2}{2}}|\nabla u_{\varepsilon}|^{2} \geq| \nabla{u_{\varepsilon}}|^{p}, \\& \bigl( \vert \nabla u_{\varepsilon} \vert ^{2}+\varepsilon \bigr)^{\frac{p - 2}{2}}|\nabla u_{\varepsilon}|\leq\bigl( \vert \nabla u_{\varepsilon} \vert ^{p-1}+1\bigr), \end{aligned}$$

and, when $1< p<2$,

$$\begin{aligned}& \bigl( \vert \nabla u_{\varepsilon} \vert ^{2}+\varepsilon \bigr)^{\frac{p - 2}{2}}|\nabla u_{\varepsilon}|^{2} \geq\bigl( \vert \nabla u_{\varepsilon} \vert ^{2}+\varepsilon\bigr)^{\frac {p}{2}}- \varepsilon^{\frac{p}{2}}, \\& \bigl( \vert \nabla u_{\varepsilon} \vert ^{2}+\varepsilon \bigr)^{\frac{p - 2}{2}}|\nabla u_{\varepsilon}|\leq\bigl( \vert \nabla u_{\varepsilon} \vert ^{2}+\varepsilon\bigr)^{\frac{p - 1}{2}}, \end{aligned}$$

then in both cases, by (2.15), we have

2.17 $$ \begin{aligned}[b] &\frac{1}{2} \iint_{Q_{T}} \psi_{t}u_{\varepsilon}^{2} \,dx\,dt - \iint_{Q_{T}} \rho_{\varepsilon}^{\alpha}u_{\varepsilon}\bigl(|\nabla u_{\varepsilon}|^{2}+\varepsilon \bigr)^{\frac{p-2}{2}}\nabla u_{\varepsilon}\cdot\nabla\psi \,dx\,dt \\ &\quad {}- \sum_{i = 1}^{N} \iint_{Q_{T}} b_{i}(u_{\varepsilon}) (u_{\varepsilon x_{i}}\psi + u_{\varepsilon}\psi_{x_{i}})\,dx\,dt + \iint_{Q_{T}}q(x)|u_{\varepsilon}|^{\gamma-1}u_{\varepsilon}\varphi \,dx\,dt \\ &\quad {}+\varepsilon^{\frac{p}{2}}c(\Omega)- \iint_{Q_{T}}\rho_{\varepsilon}^{\alpha}\psi|\nabla u_{\varepsilon}|^{p-2}\nabla u_{\varepsilon }\nabla v\,dx\,dt \\ &\quad {}- \iint_{Q_{T}}\rho_{\varepsilon}^{\alpha}\psi|\nabla v|^{p-2}\nabla (u_{\varepsilon}- v)\,dx\,dt\geq0. \end{aligned} $$

Thus

2.18 $$ \begin{aligned}[b] &\frac{1}{2} \iint_{Q_{T}} \psi_{t}u_{\varepsilon}^{2} \,dx\,dt - \iint_{Q_{T}} \rho_{\varepsilon}^{\alpha}u_{\varepsilon}\bigl(|\nabla u_{\varepsilon}|^{2}+\varepsilon \bigr)^{\frac{p-2}{2}}\nabla u_{\varepsilon}\cdot\nabla\psi \,dx\,dt \\ &\quad {}- \sum_{i = 1}^{N} \iint_{Q_{T}} b_{i}(u_{\varepsilon}) (u_{\varepsilon x_{i}}\psi + u_{\varepsilon}\psi_{x_{i}})\,dx\,dt + \iint_{Q_{T}}q(x)|u_{\varepsilon}|^{\gamma-1}u_{\varepsilon}\varphi \,dx\,dt \\ &\quad {}+\varepsilon^{\frac{p}{2}}c(\Omega)- \iint_{Q_{T}}\rho_{\varepsilon}^{\alpha}\psi|\nabla u_{\varepsilon}|^{p-2}\nabla u_{\varepsilon }\nabla v\,dx\,dt \\ &\quad {}- \iint_{Q_{T}} \psi\rho^{\alpha} \vert \nabla v \vert ^{p - 2}\nabla v \cdot(\nabla u_{\varepsilon}- \nabla v)\,dx\,dt \\ &\quad {}+ \iint_{Q_{T}} \psi\bigl(\rho^{\alpha}- \rho_{\varepsilon}^{\alpha}\bigr) \vert \nabla v \vert ^{p - 2}\nabla v \cdot(\nabla u_{\varepsilon}- \nabla v)\,dx\,dt \geqslant0. \end{aligned} $$

Notice

2.19 $$\begin{aligned}& \biggl\vert { \iint_{{Q_{T}}} \psi\bigl(\rho^{\alpha}- \rho_{\varepsilon}^{\alpha}\bigr){{ \vert {\nabla v} \vert }^{p - 2}}\nabla v \cdot(\nabla {u_{\varepsilon}} - \nabla v)\, {d}x\, {d}t} \biggr\vert \\& \quad \leqslant \sup_{(x,t) \in{Q_{T}}} \frac{{ \vert {\psi(\rho^{\alpha}- \rho_{\varepsilon}^{\alpha})} \vert }}{{\rho ^{\alpha}}} \iint_{{Q_{T}}} {\rho^{\alpha}} {{ \vert {\nabla v} \vert }^{p - 1}} \vert {\nabla{u_{\varepsilon}} - \nabla v} \vert \, {d}x \, {d}t \\& \quad \leqslant \sup_{(x,t) \in{Q_{T}}} \frac{{ \vert {\psi(\rho^{\alpha}- \rho_{\varepsilon}^{\alpha})} \vert }}{{\rho ^{\alpha}}} \biggl( \iint_{{Q_{T}}} \rho^{\alpha}{ \vert {\nabla v} \vert }^{p}{\, {d}}x\, {d}t + \iint_{{Q_{T}}} \rho^{\alpha} \vert \nabla v \vert ^{p-1} \vert \nabla{u_{\varepsilon}} \vert \, {d}x\, {d}t\biggr) \end{aligned}$$

and by the Hölder inequality

$$\begin{aligned}& \iint_{Q_{T}} \rho^{\alpha} \vert \nabla v \vert ^{p - 1} \vert \nabla u_{\varepsilon} \vert \,dx\,dt \\& \quad \leqslant \biggl( \iint_{Q_{T}} \bigl(\rho^{m} \vert \nabla v \vert ^{p - 1}\bigr)^{s}\,dx\,dt\biggr)^{1/s} \cdot\biggl( \iint_{Q_{T}} \bigl(\rho^{n} \vert \nabla u_{\varepsilon} \vert \bigr)^{p}\,dx\,dt\biggr)^{1/p}, \end{aligned}$$

where $m = \frac{{\alpha(p - 1)}}{p}$, $n = \frac{\alpha }{p}$, $s = \frac{p}{{p - 1}}$. Due to ${\rho^{\alpha}}{ \vert {\nabla u} \vert ^{p}},{\rho^{\alpha}}{ \vert {\nabla v} \vert ^{p}} \in{L^{1}}({Q_{T}})$, we have

$$\iint_{Q_{T}} \rho^{\alpha} \vert \nabla v \vert ^{p}dxdt + \iint _{Q_{T}} \rho^{\alpha} \vert \nabla v \vert ^{p - 1} \vert \nabla u_{\varepsilon} \vert \,dx\,dt \leqslant c. $$

Let $\varepsilon\rightarrow0$ in (2.19). It converges to 0.

Once more, we notice that

2.20 $$\begin{aligned}& \begin{gathered} \bigl(|\nabla u_{\varepsilon}|^{2}+\varepsilon\bigr)^{\frac{p-2}{2}} \nabla u_{\varepsilon}\\ \quad =|\nabla u_{\varepsilon}|^{p-2}\nabla u_{\varepsilon}+\frac {p-2}{2}\varepsilon \int_{0}^{1}\bigl(|\nabla u_{\varepsilon }|^{2}+ \varepsilon s\bigr)^{\frac{p-4}{2}}\, ds \nabla u_{\varepsilon}, \\ \lim_{\varepsilon\rightarrow0} \iint_{Q_{T}}\frac{p-2}{2}\varepsilon \int_{0}^{1}\bigl(|\nabla u_{\varepsilon}|^{2}+ \varepsilon s\bigr)^{\frac{p-4}{2}}\, ds \nabla u_{\varepsilon}\nabla\psi u_{\varepsilon}\,dx\,dt=0. \end{gathered} \end{aligned}$$

Let $\varepsilon\rightarrow0$ in (2.18), we have

$$\begin{aligned}& \frac{1}{2} \iint_{Q_{T}} \psi_{t}u^{2}\,dx\,dt - \iint_{Q_{T}} u\vec{\zeta}\cdot\nabla\psi \,dx\,dt - \sum_{i = 1}^{N} \iint_{Q_{T}} b_{i}(u) (u_{x_{i}}\psi + u\psi _{x_{i}})\,dx\,dt \\& \quad {}- \iint_{Q_{T}} \psi\vec{\zeta}\cdot\nabla v\,dx\,dt - \iint_{Q_{T}} \psi\rho^{\alpha} \vert \nabla v \vert ^{p - 2}\nabla v \cdot(\nabla u - \nabla v)\,dx\,dt \\& \quad {}+ \iint_{Q_{T}}q(x)|u|^{\gamma-1}u\varphi \,dx\,dt\geqslant0. \end{aligned}$$

Let $\varphi=\psi u$ in (2.13), we get

$$\begin{aligned}& \iint_{Q_{T}} \psi\vec{\zeta}\cdot\nabla u\, dx\, dt- \frac {1}{2} \iint_{Q_{T}} u^{2}\psi_{t}\, dx\, dt + \iint_{Q_{T}} u\vec{\zeta}\cdot\nabla\psi \,dx\,dt \\& \quad {}+ \sum_{i = 1}^{N} \iint_{Q_{T}} b_{i}(u) (u_{x_{i}}\psi + u\psi _{x_{i}})\,dx\,dt+ \iint_{Q_{T}}q(x)|u|^{\gamma-1}u\psi u \,dx\,dt= 0. \end{aligned}$$

Thus

2.21 $$ \iint_{{Q_{T}}} \psi\bigl(\vec{\zeta}- \rho^{\alpha}{ \vert {\nabla v} \vert ^{p - 2}}\nabla v\bigr) \cdot(\nabla u - \nabla v){\, {d}}x\, {d}t \geqslant0. $$

Let $v=u- \lambda\varphi_{1}$, $\lambda>0$, $\varphi_{1} \in C_{0}^{\infty }(Q_{T})$ is given in (2.14), then

$$\iint_{{Q_{T}}} \psi\bigl[\vec{\zeta}- \rho^{\alpha}{ \bigl\vert \nabla (u - \lambda\varphi_{1} ) \bigr\vert ^{p - 2}}\nabla (u - \lambda\varphi_{1} )\bigr] \cdot \nabla\varphi_{1} \,dx\,dt \geqslant0. $$

If $\lambda\rightarrow0$, then

$$\iint_{{Q_{T}}} \psi\bigl(\vec{\zeta}- \rho^{\alpha}{ \vert {\nabla u} \vert ^{p - 2}}\nabla u\bigr) \cdot\nabla\varphi_{1} \,dx\,dt\geqslant0. $$

Moreover, if $\lambda<0$, similarly we can get

$$\iint_{{Q_{T}}} \psi\bigl(\vec{\zeta}- \rho^{\alpha}{ \vert {\nabla u} \vert ^{p - 2}}\nabla u\bigr) \cdot\nabla\varphi_{1} \leqslant0. $$

Thus

$$\iint_{{Q_{T}}} \psi\bigl(\vec{\zeta}- \rho^{\alpha}{ \vert {\nabla u} \vert ^{p - 2}}\nabla u\bigr) \cdot\nabla\varphi_{1} \,dx\,dt= 0. $$

Noticing that $\psi= 1$ on $\operatorname{supp}\varphi_{1}$, (2.14) holds.

At same time, we are able to prove (1.9) as in [15], thus we have Theorem 1.2.

## The uniqueness without the boundary value condition

### Lemma 3.1

*Let* $u\in\mathbf{W}(Q_{T})$, $u_{t}\in\mathbf {W}'(Q_{T})$. *Then* ∀ *a*.*e*. $t_{1}, t_{2}\in(0, T)$,

3.1 $$ \int_{t_{1}}^{t_{2}} \int_{\Omega}uu_{t}\,dx\,dt=\frac{1}{2}\biggl[ \int_{\Omega }\bigl(u^{2}(x,t_{2})-u^{2}(x,t_{1}) \bigr)\, dx\biggr]. $$

This is Corollary 2.1 of [9].

### Proof of Theorem 1.3

Let *u*, *v* be two solutions of equation (1.3) with the initial values $u_{0}(x)$, $v_{0}(x)$, respectively. Denote $\Omega_{\lambda}=\{x\in\Omega: \operatorname{dist}(x,\partial\Omega )>\lambda\}$, let the constant $\beta\geq \max\{\frac{p-\alpha}{p-1}, 2, \alpha p\}$ and

3.2 $$ \xi_{\lambda}=\bigl[\operatorname{dist}(x,\Omega\setminus \Omega_{\lambda})\bigr]^{\beta }=d_{\lambda}^{\beta}. $$

We may choose $\chi_{[\tau,s]}(u_{\varepsilon}-v_{\varepsilon})\xi _{\lambda}$ as a test function, where $u_{\varepsilon}$ and $v_{\varepsilon}$ are the mollified function of the solutions *u* and *v*, respectively. Then

3.3 $$\begin{aligned}& \bigl\langle \!\bigl\langle (u-v)_{t}, \chi_{[\tau,s]}(u_{\varepsilon}-v_{\varepsilon}) \xi _{\lambda}\bigr\rangle \!\bigr\rangle \\& \quad = \iint_{Q_{\tau s}}(u_{\varepsilon}-v_{\varepsilon }) \xi_{\lambda}\frac{\partial(u-v)}{\partial t}\,dx\,dt \\& \quad =- \iint_{Q_{\tau s}}\rho^{\alpha}\bigl(|\nabla u|^{p-2} \nabla u-|\nabla v|^{p-2}\nabla v\bigr)\nabla\bigl[(u_{\varepsilon}-v_{\varepsilon}) \xi _{\lambda}\bigr] \,dx\,dt \\& \qquad {}-\sum_{i=1}^{N} \iint_{Q_{\tau s}}\bigl[b_{i}(u)-b_{i}(v)\bigr] \bigl[(u_{\varepsilon }-v_{\varepsilon})\xi_{\lambda}\bigr]_{x_{i}} \,dx\,dt \\& \qquad {}+ \iint_{Q_{\tau s}}q(x) \bigl(|u|^{\gamma-1}u-|v|^{\gamma -1}v \bigr) (u_{\varepsilon}-v_{\varepsilon})\xi_{\lambda}\,dx \,dt, \end{aligned}$$

where $Q_{\tau s}=\Omega\times(\tau, s)$. For any give $\lambda>0$, since $\nabla u\in L^{p}(\Omega_{\lambda})$, $\nabla v\in L^{p}(\Omega_{\lambda})$, according to the definition of the mollified function $u_{\varepsilon}$ and $v_{\varepsilon}$, we have

3.4 $$\begin{aligned}& u_{\varepsilon}\in L^{\infty}(Q_{T}),\qquad v_{\varepsilon} \in L^{\infty }(Q_{T}),\qquad u_{\varepsilon}\rightarrow u,\qquad v_{\varepsilon}\rightarrow v,\quad \text{a.e. in } Q_{T}, \end{aligned}$$

3.5 $$\begin{aligned}& \!\begin{aligned} &\|\nabla u_{\varepsilon}\|_{p,\Omega_{\lambda}}\leq\|\nabla u\| _{p,\Omega_{\lambda}}, \qquad \|\nabla v_{\varepsilon}\|_{p,\Omega _{\lambda}}\leq\|\nabla v \|_{p,\Omega_{\lambda}}, \\ &u_{\varepsilon }\rightharpoonup u, \qquad v_{\varepsilon}\rightharpoonup v,\quad \text{in } W^{1,p}( \Omega_{\lambda}). \end{aligned} \end{aligned}$$

Let us analyze every term in (3.3). For a start, we deal with the first term on the right hand side of (3.3). Since on $\Omega_{\lambda}$,

$$\bigl\vert \rho^{\alpha}\bigl(|\nabla u|^{p-2}\nabla u-|\nabla v|^{p-2}\nabla v\bigr) \bigr\vert \in L^{\frac{p}{p-1}}( \Omega_{\lambda}) $$

by the weak convergency of (3.5)

$$\begin{aligned}& \lim_{\varepsilon\rightarrow0} \iint_{Q_{\tau s}}\rho^{\alpha}\xi _{\lambda}\bigl(|\nabla u|^{p-2}\nabla u-|\nabla v|^{p-2}\nabla v\bigr)\nabla (u_{\varepsilon}-v_{\varepsilon}) \,dx\,dt \\& \quad = \iint_{Q_{\tau s}}\rho^{\alpha}\xi_{\lambda}\bigl(|\nabla u|^{p-2}\nabla u-|\nabla v|^{p-2}\nabla v\bigr)\nabla(u-v) \,dx \,dt. \end{aligned}$$

By (3.4)-(3.5), using the Lebesgue dominated convergence theorem,

$$\begin{aligned}& \lim_{\varepsilon\rightarrow0} \iint_{Q_{\tau s}}\rho^{\alpha }\bigl(|\nabla u|^{p-2} \nabla u-|\nabla v|^{p-2}\nabla v\bigr) (u_{\varepsilon }-v_{\varepsilon}) \nabla\xi_{\lambda} \,dx\,dt \\& \quad = \iint_{Q_{\tau s}}\rho^{\alpha}\bigl(|\nabla u|^{p-2} \nabla u-|\nabla v|^{p-2}\nabla v\bigr) (u-v)\nabla\xi_{\lambda} \,dx\,dt. \end{aligned}$$

So

3.6 $$\begin{aligned}& \lim_{\varepsilon\rightarrow0} \iint_{Q_{\tau s}}\rho^{\alpha }\bigl(|\nabla u|^{p-2} \nabla u-|\nabla v|^{p-2}\nabla v\bigr)\nabla \bigl[(u_{\varepsilon}-v_{\varepsilon}) \xi_{\lambda}\bigr] \,dx\,dt \\& \quad = \iint_{Q_{\tau s}}\rho^{\alpha}\xi_{\lambda}\bigl(|\nabla u|^{p-2}\nabla u-|\nabla v|^{p-2}\nabla v\bigr)\nabla(u-v) \,dx \,dt \\& \qquad {}+ \iint_{Q_{\tau s}}\rho^{\alpha}\bigl(|\nabla u|^{p-2} \nabla u-|\nabla v|^{p-2}\nabla v\bigr) (u-v)\nabla\xi_{\lambda} \,dx\,dt. \end{aligned}$$

We have

3.7 $$ \iint_{Q_{\tau s}}\rho^{\alpha}\xi_{\lambda}\bigl(|\nabla u|^{p-2}\nabla u-|\nabla v|^{p-2}\nabla v\bigr)\nabla(u-v) \,dx \,dt\geq0 $$

and

3.8 $$ \begin{aligned}[b] &\lim_{\lambda\rightarrow0} \biggl\vert \iint_{Q_{\tau s}}\rho^{\alpha }\bigl(|\nabla u|^{p-2} \nabla u-|\nabla v|^{p-2}\nabla v\bigr) (u-v)\nabla\xi _{\lambda} \,dx\,dt \biggr\vert \\ &\quad = \biggl\vert \iint_{Q_{\tau s}}(u-v)\rho^{\alpha}\bigl(|\nabla u|^{p-2}\nabla u-|\nabla v|^{p-2}\nabla v\bigr)\nabla \rho^{\beta}\,dx\,dt \biggr\vert \\ &\quad \leq \iint_{Q_{\tau s}}|u-v|\rho^{\alpha}\bigl(|\nabla u|^{p-1}+|\nabla v|^{p-1}\bigr)|\nabla\rho^{\beta}|\,dx \,dt \\ &\quad \leq c \biggl( \int_{\tau}^{s} \int_{\Omega}\rho^{\alpha}\bigl(|\nabla u|^{p}+| \nabla v|^{p}\bigr)\,dx\,dt \biggr)^{\frac{p-1}{p}} \cdot \biggl( \int_{\tau}^{s} \int_{\Omega}\rho^{\alpha} \bigl\vert \nabla \rho^{\beta} \bigr\vert ^{p}|u-v|^{p}\,dx\,dt \biggr)^{\frac{1}{p}} \\ &\quad \leq c \biggl( \int_{\tau}^{s} \int_{\Omega}\rho^{\alpha}\bigl(|\nabla u|^{p}+| \nabla v|^{p}\bigr)\,dx\,dt \biggr)^{\frac{p-1}{p}} \cdot \biggl( \int_{\tau}^{s} \int_{\Omega}\rho^{\alpha+p(\beta -1)}|u-v|^{p}\,dx\,dt \biggr)^{\frac{1}{p}} \\ &\quad \leq c \biggl( \int_{\tau}^{s} \int_{\Omega}\rho^{\alpha+p(\beta -1)}|u-v|^{p}\,dx\,dt \biggr)^{\frac{1}{p}}. \end{aligned} $$

Here, we have used the fact that $|\nabla\rho|=1$ is true almost everywhere. Now, by $\beta\geq\frac{p-\alpha}{p-1}$, we have

3.9 $$\begin{aligned}& \biggl\vert \iint_{Q_{\tau s}}(u-v)\rho^{\alpha}\bigl(|\nabla u|^{p-2}\nabla u-|\nabla v|^{p-2}\nabla v\bigr)\nabla \rho^{\beta}\,dx\,dt \biggr\vert \\& \quad \leq c \biggl( \int_{\tau}^{s} \int_{\Omega}\rho^{\beta }|u-v|^{p}\,dx\,dt \biggr)^{\frac{1}{p}}. \end{aligned}$$

If $p\geq2$,

3.10 $$ \biggl( \int_{\tau}^{s} \int_{\Omega}\rho^{\beta }|u-v|^{p}\,dx\,dt \biggr)^{\frac{1}{p}}\leq c \biggl( \int_{\tau}^{s} \int _{\Omega}\rho^{\beta}|u-v|^{2}\,dx\,dt \biggr)^{\frac{1}{p}}. $$

If $1< p<2$, by the Hölder inequality

3.11 $$ \biggl( \int_{\tau}^{s} \int_{\Omega}\rho^{\beta }|u-v|^{p}\,dx\,dt \biggr)^{\frac{1}{p}}\leq c \biggl( \int_{\tau}^{s} \int _{\Omega}\rho^{\beta}|u-v|^{2}\,dx\,dt \biggr)^{\frac{1}{2}}. $$

Now we deal the second term on the right hand side of (3.3). By the Lebesgue dominated convergence theorem and the Hölder inequality

3.12 $$\begin{aligned}& \lim_{\lambda\rightarrow0}\lim_{\varepsilon\rightarrow0} \iint _{Q_{\tau s}}\bigl[b_{i}(u)-b_{i}(v)\bigr] \bigl[(u_{\varepsilon}-v_{\varepsilon})\xi _{\lambda}\bigr]_{x_{i}} \,dx\,dt \\& \quad =\lim_{\lambda\rightarrow0} \iint_{Q_{\tau s}}\bigl[b_{i}(u)-b_{i}(v)\bigr] \bigl[(u-v)\xi_{\lambda}\bigr]_{x_{i}} \,dx\,dt \\& \quad =\lim_{\lambda\rightarrow0} \biggl( \iint_{Q_{\tau s}}\bigl[b_{i}(u)-b_{i}(v) \bigr](u-v)\xi_{\lambda x_{i}} \,dx\,dt \\& \qquad {}+ \iint _{Q_{s}}\bigl[b_{i}(u)-b_{i}(v) \bigr](u-v)_{x_{i}}\xi_{\lambda} \,dx\,dt \biggr) \\& \quad = \iint_{Q_{\tau s}}\bigl[b_{i}(u)-b_{i}(v) \bigr](u-v)\rho^{\beta}_{ x_{i}} \,dx\,dt+ \iint_{Q_{s}}\bigl[b_{i}(u)-b_{i}(v) \bigr](u-v)_{x_{i}}\rho^{\beta} \,dx\,dt. \end{aligned}$$

Since $\beta\geq2$, $|\rho_{x_{i}}|\leq|\nabla\rho|=1$, by the Hölder inequality,

3.13 $$\begin{aligned}& \iint_{Q_{\tau s}}\bigl[b_{i}(u)-b_{i}(v) \bigr](u-v)\rho^{\beta}_{x_{i}} \,dx\,dt \\& \quad = \int_{\tau}^{s} \int_{\Omega_{\lambda}}\bigl[b_{i}(u)-b_{i}(v) \bigr](u-v)\rho ^{\beta-1}| \rho_{x_{i}}|\, dx \\& \quad \leq \int_{\tau}^{s} \int_{\Omega}|u-v|\rho^{\beta-1}dx \leq c \biggl( \int_{\tau}^{s} \int_{\Omega}\rho^{\beta }|u-v|^{2}\,dx\,dt \biggr)^{\frac{1}{2}}. \end{aligned}$$

Since $\beta\geq\alpha p $, we have

$$\biggl(\beta-\frac{\alpha}{p}\biggr)\frac{p}{p-1}\geq\beta, $$

by this result, we have

3.14 $$\begin{aligned}& \biggl\vert \iint_{Q_{\tau s}}\bigl[b_{i}(u)-b_{i}(v) \bigr](u-v)_{x_{i}}\rho^{\beta} \,dx\,dt \biggr\vert \\& \quad \leq\sum_{i=1}^{N} \biggl( \int_{\tau}^{s} \int_{\Omega}\rho^{(\beta -\frac{\alpha}{p})p'}\bigl( \bigl\vert b_{i}(u)-b_{i}(v) \bigr\vert \bigr)^{p'}\,dx \,dt \biggr)^{\frac{1}{p'}} \\& \qquad {}\times \biggl( \int_{\tau}^{s} \int_{\Omega}\rho^{\alpha}\bigl(|\nabla u|^{p}+| \nabla v|^{p}\bigr) \,dx\,dt \biggr)^{\frac{1}{p}} \\& \quad \leq c\sum_{i=1}^{N} \biggl( \int_{\tau}^{s} \int_{\Omega}\rho^{\beta }\bigl( \bigl\vert b_{i}(u)-b_{i}(v) \bigr\vert \bigr)^{p'}\,dx \,dt \biggr)^{\frac{1}{p'}} \\& \quad \leq c \biggl( \int_{\tau}^{s} \int_{\Omega}\rho^{\beta }|u-v|^{p'}\,dx\,dt \biggr)^{\frac{1}{p'}}. \end{aligned}$$

If $p>2$, then $1< p'<2$. By the Hölder inequality,

3.15 $$ \biggl( \int_{\tau}^{s} \int_{\Omega}\rho^{\beta }|u-v|^{p'}\,dx\,dt \biggr)^{\frac{1}{p'}} \leq c \biggl( \int_{\tau}^{s} \int_{\Omega}\rho^{\beta }|u-v|^{2}\,dx\,dt \biggr)^{\frac{1}{2}}, $$

If $1< p\leq2$, then $p'\geq2$,

3.16 $$ \biggl( \int_{\tau}^{s} \int_{\Omega}\rho^{\beta }|u-v|^{p'}\,dx\,dt \biggr)^{\frac{1}{p'}} \leq c \biggl( \int_{\tau}^{s} \int_{\Omega}\rho^{\beta }|u-v|^{2}\,dx\,dt \biggr)^{\frac{1}{p'}}. $$

Again, for the third term on the right hand side of (3.3),

3.17 $$\begin{aligned}& \lim_{\lambda\rightarrow0}\lim_{\varepsilon\rightarrow0} \iint _{Q_{\tau s}}q(x) \bigl(|u|^{\gamma-1}u-|v|^{\gamma-1}v \bigr) (u_{\varepsilon }-v_{\varepsilon})\xi_{\lambda}\,dx\,dt \\& \quad \leq \iint_{Q_{\tau s}}q(x) \bigl\vert u^{\gamma}-v^{\gamma} \bigr\vert |u-v|\rho^{\beta }\,dx\,dt\leq c \iint_{Q_{\tau s}}q(x)\rho^{\beta}|u-v|\,dx\,dt \\& \quad \leq c \iint_{Q_{\tau s}}\sqrt{\rho^{\beta}}|u-v|\,dx\,dt\leq c \biggl( \int _{\tau}^{s} \int_{\Omega}\rho^{\beta}|u-v|^{2}\,dx\,dt \biggr)^{\frac {1}{2}}. \end{aligned}$$

At last, by Lemma 3.1,

3.18 $$\begin{aligned}& \lim_{\lambda\rightarrow0}\lim_{\varepsilon\rightarrow0} \iint _{Q_{\tau s}}(u_{\varepsilon}-v_{\varepsilon}) \xi_{\lambda}\frac {\partial(u-v)}{\partial t}\,dx\,dt \\& \quad =\lim_{\lambda\rightarrow0} \iint_{Q_{\tau s}}(u-v)\sqrt{\xi _{\lambda}}\frac{\partial\sqrt{\xi_{\lambda}}(u-v)}{\partial t} \,dx\,dt \\& \quad =\frac{1}{2}\lim_{\lambda\rightarrow0} \int_{\Omega}\xi_{\lambda }\bigl[(u-v)^{2}(x,s)-(u-v)^{2}(x, \tau)\bigr]\, dx \\& \quad =\frac{1}{2}\biggl\{ \int_{\Omega}\rho^{\beta}\bigl[u(x,s)-v(x,s) \bigr]^{2}\, dx- \int _{\Omega}\rho^{\beta}\bigl[u(x,\tau)-v(x,\tau) \bigr]^{2}\, dx\biggr\} . \end{aligned}$$

Now, after letting $\varepsilon\rightarrow0$, let $\lambda \rightarrow0$ in (3.3). Then, by (3.7)-(3.18),

3.19 $$\begin{aligned}& \int_{\Omega}\rho^{\beta}\bigl[u(x,s)-v(x,s) \bigr]^{2}\, dx- \int_{\Omega}\rho ^{\beta}\bigl[u(x,\tau)-v(x,\tau) \bigr]^{2}\, dx \\& \quad \leq c \biggl( \int_{\tau}^{s} \int_{\Omega}\rho^{\beta } \bigl\vert u(x,t)-v(x,t) \bigr\vert ^{2}\,dx\,dt \biggr)^{k}, \end{aligned}$$

where $k<1$. By this inequality, we are able to show that

3.20 $$ \int_{\Omega}\rho^{\beta} \bigl\vert u(x,s)-v(x,s) \bigr\vert ^{2}\, dx\leq \int_{\Omega}\rho^{\beta} \bigl\vert u(x,\tau )-v(x,\tau) \bigr\vert ^{2}\, dx. $$

Thus, by the arbitrariness of *τ*, we have

3.21 $$ \int_{\Omega}\rho^{\beta} \bigl\vert u(x,s)-v(x,s) \bigr\vert ^{2}\, dx\leq \int _{\Omega}\rho^{\beta} \vert u_{0}-v_{0} \vert ^{2} \, dx. $$

By (3.21), we clearly have (1.10). The proof is complete. □

## Conclusion

The equations considered in this paper come from many applied fields such as mechanics, biology, etc. The main points of focus of this paper are two aspects. One is that the weak solution defined in this paper satisfies $u_{t}\in W'(Q_{T})$, then the uniqueness can be proved. The other one is to show that the degeneracy of the diffusion coefficient $\rho^{\alpha}$ can take place with the usual boundary value condition.
